# Genome-wide transcriptomic profiling of *Anopheles gambiae *hemocytes reveals pathogen-specific signatures upon bacterial challenge and *Plasmodium berghei *infection

**DOI:** 10.1186/1471-2164-10-257

**Published:** 2009-06-05

**Authors:** Luke A Baton, Anne Robertson, Emma Warr, Michael R Strand, George Dimopoulos

**Affiliations:** 1 W. Harry Feinstone Department of Molecular Microbiology & Immunology, Malaria Research Institute, Bloomberg School of Public Health, Johns Hopkins University, 615 N Wolfe Street, Baltimore, MD 21205-2179, USA; 2 Department of Entomology and Center for Emerging and Tropical Global Diseases, University of Georgia, Athens, GA 30602-2603, USA; 3 Current address: Centre for Applied Entomology and Parasitology, School of Life Sciences, Huxley Building, Keele University, Staffordshire, ST5 5BG, UK

## Abstract

**Background:**

The mosquito *Anopheles gambiae *is a major vector of human malaria. Increasing evidence indicates that blood cells (hemocytes) comprise an essential arm of the mosquito innate immune response against both bacteria and malaria parasites. To further characterize the role of hemocytes in mosquito immunity, we undertook the first genome-wide transcriptomic analyses of adult female *An. gambiae *hemocytes following infection by two species of bacteria and a malaria parasite.

**Results:**

We identified 4047 genes expressed in hemocytes, using *An. gambiae *genome-wide microarrays. While 279 transcripts were significantly enriched in hemocytes relative to whole adult female mosquitoes, 959 transcripts exhibited immune challenge-related regulation. The global transcriptomic responses of hemocytes to challenge with different species of bacteria and/or different stages of malaria parasite infection revealed discrete, minimally overlapping, pathogen-specific signatures of infection-responsive gene expression; 105 of these represented putative immunity-related genes including anti-*Plasmodium *factors. Of particular interest was the specific co-regulation of various members of the Imd and JNK immune signaling pathways during malaria parasite invasion of the mosquito midgut epithelium.

**Conclusion:**

Our genome-wide transcriptomic analysis of adult mosquito hemocytes reveals pathogen-specific signatures of gene regulation and identifies several novel candidate genes for future functional studies.

## Background

Insect blood cells (hemocytes) play a central role in mediating innate immune responses [1-4]. Hemocytes participate in defense against invading microorganisms either directly through cellular mechanisms like phagocytosis or indirectly through secretion of soluble humoral factors such as antimicrobial peptides, complement-like proteins and components of the proteolytic cascade that regulates melanization [5-12].

Much of our knowledge of insect hemocytes derives from studies with the model insect *Drosophila melanogaster *and Lepidoptera [13-17], but increasing evidence also implicates hemocytes as being essential to the immune response of mosquitoes including *Anopheles gambiae *that vectors human malaria [18,19]. In adult mosquitoes, hemocytes mediate phagocytic and/or melanotic immune responses [20-25], and express several immunity-related molecules implicated in defense against bacteria and/or malaria parasites [20-23,25-39]. As a first step in the functional genomic analysis of mosquito hemocytes, we conducted a genome-wide microarray-based transcriptomic profiling of *Anopheles gambiae *hemocytes in response to infection by bacteria and *Plasmodium berghei*. We placed particular emphasis on genes with putative functions in the mosquito's immune system.

## Results and Discussion

### The hemocyte transcriptome

We used our previously published "high injection/recovery" method to isolate hemocyte samples with little or no contamination by other cell types from adult female mosquitoes [31]. This approach results in recovery of the three types of hemocytes- granulocytes, oenocytoids and prohemocytes – produced by *An. gambiae *that are distinguished from one another by a combination of morphological, functional, and molecular characters [31].

We analyzed the transcriptional profiles of hemocytes using custom-made 60-mer oligonucleotide microarrays representing the approximately 13,100 genes of the predictedtranscriptome of *An. gambiae *[40].

In order to identify hemocyte-specific and immune-responsive transcripts, we first compared transcripts expressed in hemocytes from one day old sugar-fed mosquitoes to transcripts detected in whole mosquitoes of the same age and feeding status. This resulted in identification of the hemocyte-enriched transcriptome. We then compared hemocytes from 1 day old mosquitoes, 1 hour after immune challenge with heat-killed *Escherichia coli *or *Micrococcus luteus*, to control female mosquitoes injected with sterile PBS to determine the bacteria challenge responsive transcriptomes. We used heat-killed bacteria in these assays, because our primary interest was in identifying the bacterial responsive transcriptome and to avoid the potentially confounding effects of altered gene expression due to the lethal effects of a systemic infection associated with injection of living bacteria. Lastly, we compared hemocytes from mosquitoes at 24 hours and 19 days after ingestion of a blood meal infected with *Plasmodium berghei *to mosquitoes of the same age fed a non-infected blood meal to determine the ookinete and sporozoite infection responsive transcriptomes, respectively. This design resulted in a total of five experimental treatments.

**Figure 1:**
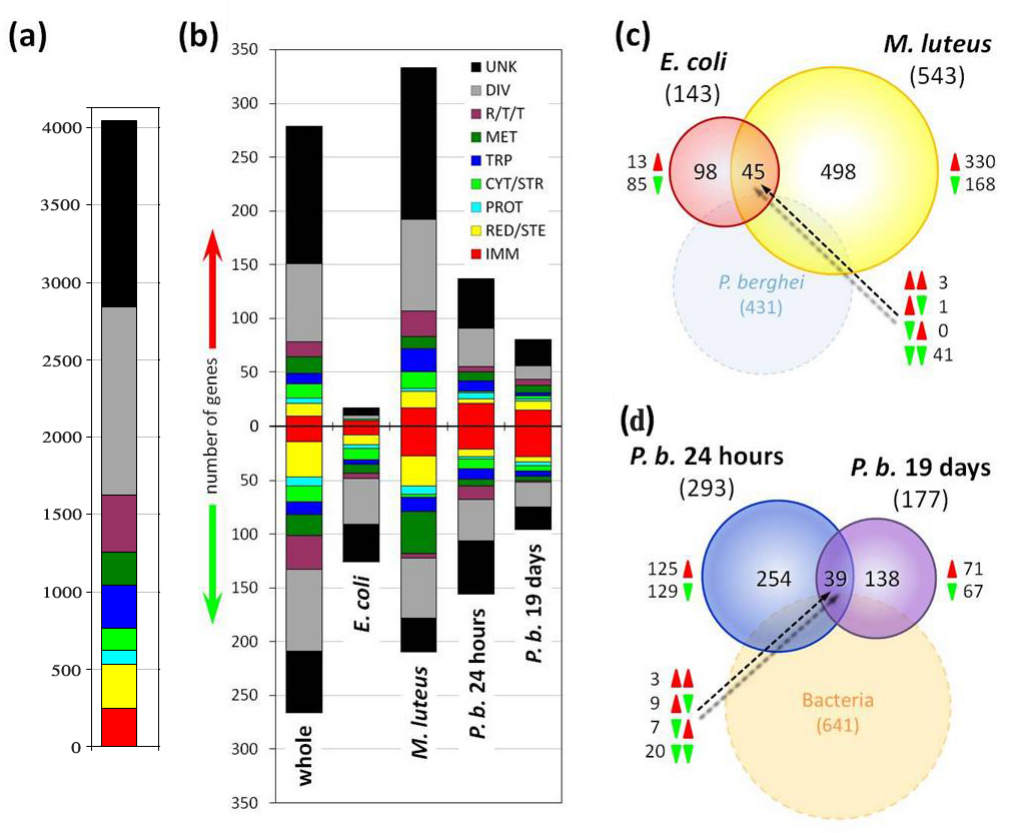
**The global transcriptomic profiles of adult female *An. gambiae *hemocytes**. **(a)**Bar chart show the gene functional group distribution of all genes for which any transcription was detected in *An. gambiae *hemocytes, in at least 1 of the 5 experimental comparisons. **(b) **Bar chart showing the number and functional class of all the genes either enriched or under-represented in hemocytes relative to whole adult female mosquitoes (whole), or significantly differentially regulated in hemocytes either following challenge with heat-killed bacteria (*E. coli *and *M. luteus*) or infected with different stages of the rodent malaria parasite *P. berghei *(*P. b*. 24 hours and *P. b*. 19 days). Red and green arrows indicate, respectively, genes up- and down-regulated in hemocytes relative to either whole adult females (whole) or hemocytes from naïve control mosquitoes (E. coli, M. luteus, *P. b*. 24 hours and *P. b*. 19 days). **(c) **and **(d) **Proportional-area Venn diagrams illustrating the distribution of genes significantly differentially transcribed in hemocytes following either bacterial challenge (c) or different stages of infection with the malaria parasite *P. berghei *(d). Numbers in brackets outside circles indicate the total number of genes differentially regulated by each species, or stage, of pathogen. Numbers in brackets inside circles indicate the pooled total number of genes differentially regulated by either both stages of malaria parasite infection (c) or both species of bacteria (d). Numbers by red and green triangles indicate the number of genes up- and down-regulated, respectively, within each segment of the three segments formed by the two upper-most circles of each figure. IMM = immunity-and apoptosis-related; RED/STE = redox and oxidoreductive stress; PROT = proteolysis; CYT/STR = cytoskeletal and structural; TRP = transport; MET = metabolism; R/T/T = replication, transcription and translation; DIV = diverse; and UNK = unknown.

Overall, we detected a total of 4047 genes expressed in hemocytes expressed in at least one of the experimental treatments (Figure 1a; Additional file 1). Only 562 (13.9%) transcripts were detected in all experimental treatments, but 2205 (54.5%) transcripts were identified in at least two of the treatments performed. We observed some variability in detectable transcript levels between the different experimental treatments that most likely reflected differences in age, infection state or other physiological factors (i.e. sugar- versus blood-fed) (see below).

Comparing gene expression between hemocytes and whole adult female mosquitoes, we identified 279 gene transcripts with at least a 2 – fold higher presence and 266 genes with a lower abundance in hemocytes compared to whole adult female mosquitoes (Figure 1b, Additional file 1). Only 54.5% of the hemocyte enriched transcripts had predicted functions and these are discussed in Additional file 2, data section S1.

### Hemocyte immune gene expression

We identified expression of 182 (54.3%) of the 335 predicted immunity-related genes in at least one of our five experimental comparisons, reinforcing the major role of hemocytes in immune defense [41,42]. Importantly and consistent with previous studies, we detected transcription of all 11 immunity-related genes previously reported to be expressed in *An. gambiae *hemocytes. These genes are presented in the Additional file 2, data section S2. Contrary to expectation, however, only 9 of the 279 transcripts with higher abundance in hemocytes versus whole mosquitoes and 14 of the 266 transcripts with lower abundance were immunity-related genes. These genes are presented in detail in the Additional file 2, data section S3.

Among the pattern recognition receptors expressed in hemocytes, FBNs, PGRPs, TEP and TOLL family members were especially well represented. In contrast, relatively fewer C-type lectins (CTLs), galectins, Gram-negative binding proteins (GNBPs) and CD36-like scavenger receptors (SCRs) were transcribed in hemocytes. Seventeen members of the leucine rich repeat (LRR) family were transcribed in hemocytes, including two of the three previously reported to mediate anti-*Plasmodium *immune responses (LRIM and LRRD7 [APL2]) [43-45]. We also detected transcription of several members of the recently characterized Nimrod superfamily, including putative homologues of Eater and NimC-1 [46] that are expressed in hemocytes from *Drosophila melanogaser *and that are involved in phagocytosis of bacteria and apoptotic cells [46]. As many as 27 CLIP-domain serine proteases, 11 of their associated serpin inhibitors [47,48], and 8 putative target PPO zymogens were also expressed in hemocytes [49,50]. All of these factors have been predicted or are experimentally implicated in melanization reactions that occur in response to infection by bacteria and *Plasmodium *[28,51]. Thirty-four other genes predicted to encode serine proteases were also transcribed in hemocytes, of which 13 were enriched in hemocytes and/or exhibited differential expression following microbial exposure.

In addition to canonical immunity-related genes, we detected several gene transcripts in hemocytes belonging to the cytoskeletal/structural functional class that have conserved roles in phagocytosis [52,53]. These genes were dominated by factors involved in the biogenesis and organization/rearrangement of the actin cytoskeleton, and included 5 components of the Arp2/3 actin-nucleation complex; 3 vesicle trafficking ADP-ribosylation factors (ARFs); cofilin; various members of the Rho, Rac and Rab families of small GTPases (including rho1/L, rac1/2, cdc42, Rab5, and several SNAREs); and several WASP family members (including SCAR) [54-56].

Comparison of our microarray expression data to EST data from bacteria-challenged hemocytes of the mosquitoes *Aedes aegypti *and *Armigeres subalbatus *[38] revealed a relatively high degree of conservation in gene transcripts associated with pathogen recognition and humoral immune responses. Conservation of hemocyte-specific transcript expression between the three mosquito species was particularly high for the following groups of immunity-related genes: antimicrobial peptides (8 of 8 expressed in *An. gambiae*); PRRs (9 of 9); CLIP-domain serine proteases and their serpin inhibitors (15 of 17); melanization (6 of 7); antioxidant-related (12 of 14); and apoptosis-related (14 of 17). In contrast, there was lower concordance between the three mosquito species in gene transcripts associated with functions related to the cytoskeleton (12 of 32), signal transduction (22 of 36) and stress responses (7 of 16). We also compared our microarray expression data to transcriptional profiles for hemocytes from larval stage *D. melanogaster *[15,16]. This analysis suggested relatively weak conservation in hemocyte gene expression (data not shown) which may reflect differences in hemocytes from different developmental stages (adults versus larvae) and the apparent absence in mosquitoes of orthologs for many hemocyte-specific *Drosophila *genes (data not shown).

### Hemocyte abundance in response to immune challenge

**Figure 2:**
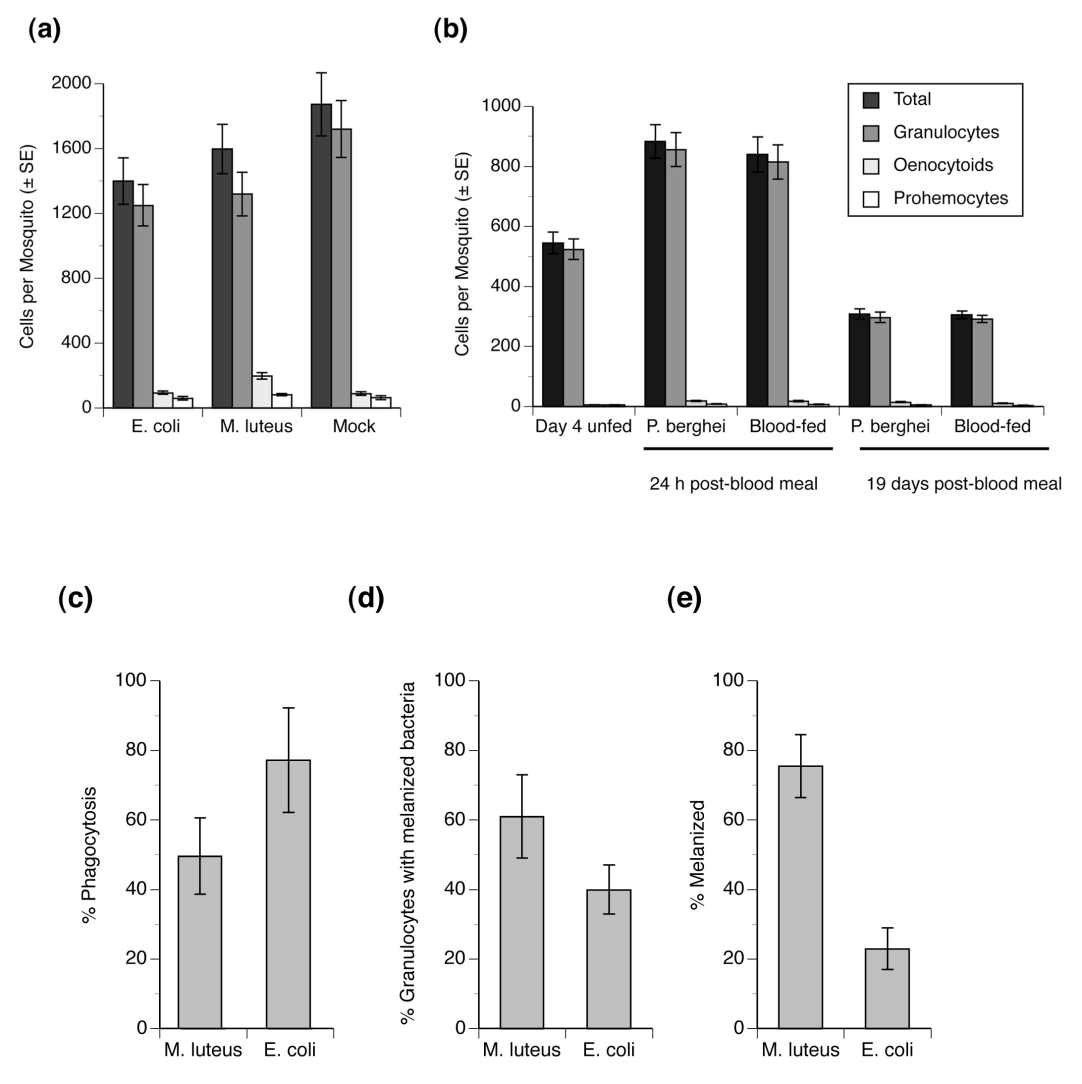
**Total number of hemocytes and hemocyte types (granulocytes, oenocytoids and prohemocytes) in *An. gambiae***. **(a) **Total number of hemocytes and abundance of hemocyte types (granulocytes, oenocytoids, or prohemocytes) per mosquito 24 h post-injection with *E. coli*, *M. luteus*, or PBS (mock). **(b) **Total number of hemocytes and abundance of hemocyte types in day 4 non-fed mosquitoes, and mosquitoes (24 h or 19 days) after feeding on a blood meal containing *P berghei *or non-infected blood meal. **(c) **Influence of bacterial species (*E. coli *or *M. luteus*) on phagocytosis by *An. gambiae *granulocytes. **(d) **Influence of bacterial species on the proportion of granulocytes with internalized bacteria that are melanized. **(e) **Influence of bacterial species on the proportion of melanized oenocytoids. A minimum of 10 mosquitoes were bled per treatment. Results for (a) and (b) are presented as means ± SE. Results for (c-e) are given as means ± SE for phagocytic or melanized cells relative to the total number of cells (100) counted per sample.

As previously noted, *An. gambiae *produces three hemocyte types with granulocytes accounting for greater than 90% of the total number of cells in circulation during the larval, pupal and adult stage [31]. In adults, however, the total number of hemocytes in circulation declines with mosquito age while blood feeding stimulates a transient increase in circulating hemocytes [31]. We reasoned that infection could also affect hemocyte abundance, which together with age or blood feeding could create variation in microarray expression ratios unrelated to differential gene regulation *per se *between control and microbe-exposed mosquitoes. To facilitate interpretation of our transcriptome data, therefore, we assessed the effects of microbial challenge on hemocyte abundance in *An. gambiae *relative to non-infected controls. Sample analysis 24 h post-infection revealed no significant differences in the total number of hemocytes in circulation (F_2,29 _= 2.09; *P *= 0.14) or in the number of granulocytes (F_2,29 _= 2.95; *P *= 0.07) and prohemocytes (F_2,29 _= 1.30; *P *= 0.29) between mosquitoes injected with *E. coli, M. luteus *or PBS (control) (Figure 2a). However, injection of *M. luteus *did induce a significant increase in the number of oenocytoids (F_2,29 _= 18.78; *P *< 0.0001) (Fig. 2a); an alteration that resulted in this hemocyte type comprising 12% of the total number of cells in circulation in *M. luteus*-challenged mosquitoes compared to 7% and 5% for *E. coli-*infected mosquitoes and PBS controls.

We infected mosquitoes with *P. berghei *by blood feeding 4 day old mosquitoes and then collecting samples 24 h or 19 days later. At the 24 h time point, parasites were in the ookinete stage in the midgut epithelium of the mosquito while at day 19 parasites were in the sporozoite stage and were detected in the salivary glands. Controls consisted of hemocytes collected from 4 day old, non-blood fed mosquitoes, and hemocytes collected 24 h and 19 days after mosquitoes fed on a non-infected bloodmeal. Consistent with previous results [31], the total number of hemocytes in circulation significantly differed among treatments (F_4,49 _= 45.86; *P *< 0.0001) with 24 h post-blood fed mosquitoes having more hemocytes in circulation than day 4 non-blood fed mosquitoes or day 19 post-blood feeding mosquitoes (Figure 2b). However, no significant differences were detected in the total number of hemocytes and hemocyte types in circulation between infected and control mosquitoes at 24 h or 19 days. Taken together, these results indicate that variation in hemocyte abundance likely affects transcript levels among the five experimental treatments we performed. Within a given treatment, however, the lack of differences in hemocyte abundance between experimental and control samples indicates that any differences in microarray expression ratios reflect differential gene expression in response to the pathogen.

This finding is important because global transcriptomic profiles revealed a remarkable degree of specificity with regard to pathogen and stage of immune challenge. Of the 4047 hemocyte transcripts detected in hemocytes, 959 (23.7%) exhibited differential regulation in at least one of the 4 experimental comparisons involving challenge by bacteria or *P. berghei *(Figure 1b–d; Additional file 1). Among these differentially expressed transcripts, immunity-related genes were significantly over-represented compared to the genes belonging to other functional classes (χ^2 ^= 27.11, *P *< 0.0001). While immunity-related genes comprised only 6.1% (247/4047) of all genes expressed in hemocytes, 10.9% (105/959) of differentially regulated transcripts belonged to this functional class. In contrast, the replication/transcription/translation functional class was significantly under-represented in the group of differentially expressed genes (χ^2 ^= 14.19, *P *= 0.0002), presumably indicative of the house-keeping function of many of the genes in this category. The percentage of differentially regulated transcripts was not significantly different from that expected under the assumption of no association between functional class and differential regulation upon challenge for the remaining 7 functional classes of genes (χ^2 ^= 5.49, *P *= 0.356).

**Figure 3:**
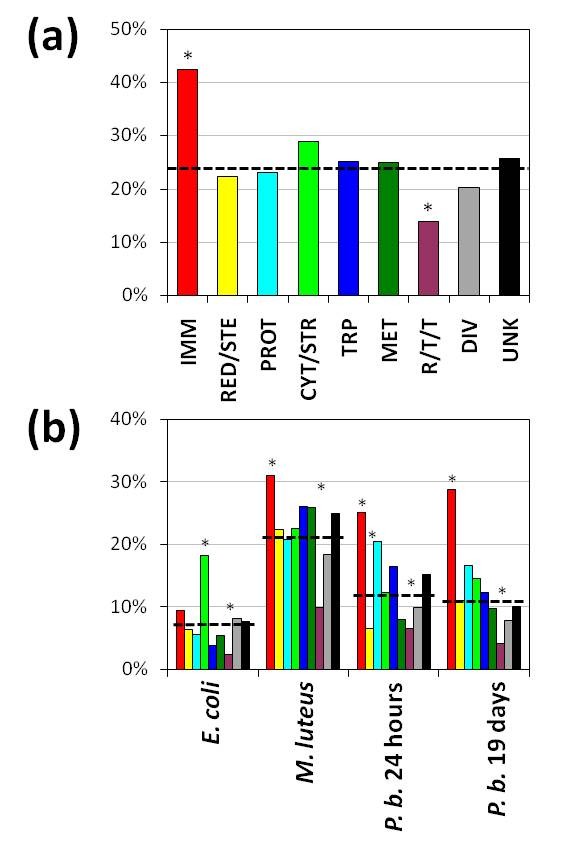
**Comparison of differential transcription according to functional class – specific responses**. The number of differentially regulated transcripts within each functional class is expressed as a percentage of the total number transcripts detected in hemocytes for each experimental comparison. **(a) **The percentage of genes, for each functional class, expressed in hemocytes which were differentially regulated following either bacterial challenge and/or malaria parasite infection. Dashed line indicates the overall percentage of each functional class in the differentially expressed genes in hemocytes; this percentage of transcripts is expected to be differentially expressed within each functional class if there is no association between functional class and differential transcription. Functional classes exhibiting significant over- or under-representation among expressed transcripts are indicated by asterisks (χ^2^-test; see main text for details). **(b) **The same as (a) but for each challenge. IMM = immunity-and apoptosis-related; RED/STE = redox/stress/mitochondrial; PROT = proteolysis; CYT/STR = cytoskeletal/structural; TRP = transporters; MET = metabolism; R/T/T = replication/transcription/translation; DIV = diverse; and UNK = unknown.

Further insight into the role of different functional classes of genes was provided by calculating the percentage of differentially expressed transcripts within each functional class for different microbial exposures (Figure 3). This analysis highlighted variation in the overall levels of differential gene transcription among treatments and pathogen-specific functional class responses. For example, in hemocytes from mosquitoes challenged with *E. coli*, the cytoskeletal/structural class was significantly over-represented in the group of differentially regulated genes compared to other functional classes (χ^2 ^= 8.32, *P *= 0.0039), while immunity-related genes were not. This likely reflects an important role for phagocytosis and hemocyte migration in defense against bacteria (χ^2 ^= 0.63, *P *= 0.298). In contrast, hemocytes from mosquitoes infected *P. berghei *exhibited an under-representation of redox and oxidoreductive stress class genes at 24 h post-infection (i.e. day 5) (χ^2 ^= 3.87, *P *= 0.049). A relatively high percentage of genes belonging to the proteolysis class were also differentially regulated although this difference was not statistically significant (χ^2 ^= 1.30, *P *= 0.255 for 24 hours post-infection and χ^2 ^= 0.78, *P *= 0.377 for 19 days p.i.).

### Transcriptional profile of hemocytes from bacteria-infected *An. gambiae*

Challenge with either heat-killed *E. coli *or *M. luteu*s resulted in the differential regulation of 641 transcripts (Figure 1b, c; Additional file 1), while only 44 transcripts (6.9%) exhibited similar regulation upon challenge with both elicitors. Challenge with *M. luteus *regulated 3.8 times more genes compared to challenge with *E. coli*: 543 genes were differentially regulated by *M. luteus*, while only 143 were regulated by *E. coli*. The percentage of expressed genes differentially regulated was also much greater for *M. luteus *than for challenge with *E. coli *(21.4% versus 7.2% of transcribed genes, respectively). This difference in gene regulation was primarily due to a lack of transcriptional up-regulation following challenge with *E. coli*: only 17 genes were induced by this bacterial species compared to 334 by *M. luteus*.

In total, 52 immunity-related genes exhibited significantly different transcription following bacterial challenge. Thirteen and 44 immunity-related genes, respectively, were differentially transcribed following challenge with either *E. coli *or *M. luteu*s (5 and 17 up- and 8 and 27 down-regulated). Only 4 (7.7%) genes differentially transcribed following bacterial challenge were comparably regulated by *E. coli *and *M. luteus*, and these are discussed in a greater detail in the Additional file 3, data section S4 (Figure 1b, c; Additional file 1). *M. luteus *challenge resulted in the up-regulation of 5 genes previously identified during an *in vivo *screen for factors in *An. gambiae *associated with phagocytosis: *cactus*, *CED6L*, *PGRPLA*, *PGRPLC *and *TEP3 *[25]. For more details on this expression signature see Additional file 2, data section S5. Four genes encoding protein products with putative roles in melanization were transcriptionally up-regulated in hemocytes following bacterial challenge, and are discussed in Additional file 2, data section S6. A number of other genes belonging to diverse functional classes and previously implicated in phagocytosis [54-59] were also differentially regulated upon bacterial challenge and most of them showed distinct patterns of transcriptional regulation for the two bacterial species and are discussed in a greater detail in the Additional file 2, data section S7.

### *An. gambiae *hemocytes differentially respond to *E. coli *and *M. luteus*

Previous studies with the mosquitoes *Anopheles albimanus*, *Aedes aegypti *and *Armigeres subalbatus *suggest that *E. coli *is primarily phagocytosed by hemocytes, while *Micrococcus *spp. are melanized extracellularly within the hemolymph [20-22]. Our own results (see above) noted a significant increase in the abundance of oenocytoids, which constitutively express phenoloxidase activity, following infection by *M. luteus*. Prior studies with *An*. *gambiae *in contrast indicate that granulocytes are the only hemocytes that phagocytize foreign targets and also inducibly express phenoloxidase activity following immune challenge by bacteria [31]. We therefore characterized phagocytosis and melanization toward *E. coli *or *M. luteus *in *An. gambiae *to assess whether: 1) oenocytoids and granulocytes differentially respond to these two bacterial species and 2) whether this response is qualitatively consistent with transcriptomic profiles. Phagocytosis assays revealed that significantly more granulocytes phagocitized *E. coli *than *M. luteus *(t-test; P < 0.01) (Figure 2c). However, we also noted that a higher proportion of granulocytes with internalized *M. luteus *contained bacteria that were melanized compared to granulocytes with internalized *E. coli *(t-test; P < 0.01) (Figure 2d). Although oenocytoids cannot phagocytize bacteria, challenge with *M. luteus *also induced a significantly greater proportion of these hemocytes to melanize than *E. coli *(t-test; P < 0.01) (Figure 2e). These results indicate that similar to other mosquitoes, *M. luteus *induces a much stronger melanization response than *E. coli *in *An. gambiae*, even though granulocytes phagocytize both species of bacteria. Although broadly consistent with the up-regulation of phagocytosis and melanization-related genes following *M. luteus *challenge, these results also do not explain why so few genes with phagocytic or immune functions are up-regulated by *E. coli*.

### Transcriptional profile of hemocytes from *Plasmodium*-infected *An. gambiae*

During the two major spatial transition stages of infection by *Plasmodium *sp., ookinete invasion of the midgut and sporozoite migration through the hemolymph, the parasite experiences considerable loss of abundance in *An. gambiae *[60,61] that is in part attributed to hemocyte-mediated immune responses [28,32]. Overall, transcripts of 431 genes were differentially expressed in hemocytes during malaria parasite infection (either at 24 hours and/or 19 days after *P. berghei *infection) (Figure 1b and 1d; Additional file 1). When considered relative to the total number of expressed genes, the magnitude of gene regulation was similar for the two different stages of malaria parasite infection (12.1 versus 11.0% of transcribed genes were differentially expressed, respectively, at 24 hours and19 days after *P. berghei *infection). Strikingly, only 23 (5.3%) of the differentially regulated transcripts had similar expression profiles during the two infection stages, while 16 (3.7%) transcripts were expressed in opposite directions. The remaining 392 (91.0%) transcripts were differentially expressed exclusively during one of the two stages of parasite infection. Only 5 (6.8%) of the 74 putative immune genes were regulated in the same direction while 7 (9.5%) transcripts were regulated in opposite directions at 24 hours and 19 days after *P. berghei *infection.

### Hemocyte transcription during *P. berghei *ookinete invasion of the midgut epithelium

At 24 hours after infection with *P. berghei*, 293 genes exhibited differential transcription in hemocytes, with 137 being up-regulated and 156 down-regulated (Figure 1b and 1d; Additional file 1). This included 42 immunity-related genes, and 3 other genes involved in lipid transport which are of particular interest because of their previously reported effects on malaria parasite infection; the retinoid and fatty-acid binding glycoprotein (*RFABG*), which encodes apolipophorins I and II of the insect lipid transporter [62]; apolipophorin III [63]; and an apolipoprotein D (ApoD; ENSANGT00000010586). *RFABG *transcription has previously been reported to be induced in the midgut epithelium during *P. berghei *ookinete invasion, and RNAi-mediated silencing of this gene significantly increases malaria parasite infection [62]. Another immune-responsive ApoD (ENSANGT00000028106), which was also expressed in hemocytes, is necessary for antibacterial and defense against at least some species of *Plasmodium *[64,65].

Although transcribed in hemocytes, we did not detect differential transcription of the majority of PRRs previously implicated in immune responses against *Plasmodium *sp.: AgMDL1, CTL4, CTLMA2, LRIM1, LRRD7 (APL2), LRRD19 (APL1), and TEP1 [28,51,64,66]. This was especially surprising for *CLT4*, *LRIM1 *and *TEP1*, which are known to be induced 24 hours after *P. berghei *infection [28,32,51]. However, transcripts encoding the PPRs FBN9 and DSCAM, which have are also implicated in defense against *P. berghei *and the human malaria parasite *P. falciparum *[64,67], were significantly up-regulated (data not shown).

Four members of the Imd/REL2 pathway were up-regulated (*IAP2*, *TAK1*, *IKK2 *and *REL2*), and one member was down-regulated (*Imd*). The Imd/REL2 pathway has previously been reported to limit *P. berghei *oocyst infection in *An. gambiae *[68], although others have been unable to replicate this finding [32]. In *Drosophila*, cross-regulation between the Imd/Relish and JNK signaling pathways is well-established [43-45,69-71] (This expression signature is discussed in detail in Additional file 2, data section S8). Nine genes encoding factors predicted to belong to the proteolytic cascades regulating melanization were differentially transcribed in hemocytes 24 hours after *P. berghei *infection and included serine proteases and their serpin inhibitors (Additional file 1). (This expression signature is discussed in detail in Additional file 2, data section S9). Four other factors with known or putative roles in melanization defense reactions were differentially transcribed in hemocytes during the period of ookinete invasion of the midgut epithelium. LYSC1 was significantly down-regulated in hemocytes at both 24 hours and 19 days after *P. berghei *infection, as well as following challenge with *M. luteus*. LYSC1 has previously been reported to be induced by bacterial challenge [72], and to inhibit melanization of Sephadex beads through interfering with PO activity [73]. Three enzymes implicated in melanogenesis were transcriptionally up-regulated, including: phenylalanine hydroxylase (PAH; also known as phenylalanine 4-monooxygenase, EC 1.14.16.1), tryptophan 2,3-dioxygenase (TO; EC 1.13.13.11) and dopamine *N*-acetyltransferase (DAT; EC 2.3.1.87). [72,74-77]. The expression of TO in hemocytes, and its significant differential expression during both ookinete invasion of the midgut epithelium and following challenge with *M. luteus *suggest a role for TO in melanotic defense reactions. DAT has not previously been implicated in insect melanization reactions, but its substrate dopamine is an intermediate in melanin production suggesting a potential role in the biochemical pathways mediating melanization.

### Hemocyte transcription during *P. berghei *sporozoite migration through the hemolymph

At 19 days after *P. berghei *infection, 177 genes were differentially transcribed in hemocytes, of which 81 were up-regulated and 96 were down-regulated (Figure 1b, d; Additional file 1). This included 43 immunity-related genes of which 28 were repressed and 15 were induced. Notably, 16 (37.2%) of the immunity-related genes differentially regulated during sporozoite migration through the hemolymph belonged to the FBN family of immunolectins: 9 FBNs were down-regulated, while 7 uncharacterized FBNs were up-regulated. The role of FBNs in anti-sporozoite defense has not been investigated, but the discrete patterns of FBN expression observed at 24 hours and 19 days after *P. berghei *infection suggests that distinct FBN subsets are involved in mosquito immune responses to ookinetes and sporozoites. The remaining 8 immunity-related genes transcriptionally up-regulated during the period of sporozoite presence in the hemolymph were: the putative MD-2-like lipid-receptor *AgMDL13*, *TEP3*, the *An. gambiae *ortholog of the *Drosophila *scavenger receptor croquemort (*SCRBQ2*), the CLIP-domain serine proteases *CLIPB8 *and *CLIPB13*, the serpins *SRPN9 *and *SRPN17*, and a thioredoxin peroxidase (*TPX4*). The role of these factors in infection by *Plasmodium *sp. has not been investigated, except for CLIPB8 which has been shown to promote melanization of ookinetes during invasion of the midgut epithelium and foreign bodies such as Sephadex beads inoculated into the thorax [78,79]. *CLIPB8*, *CLIPB13*, *SRPN9 *and *CLIPA12*, are all specifically regulated by sporozoite infection and it is tempting to speculate that are part of a common mechanism.

The 19 immunity-related genes exhibiting significant down-regulation during sporozoite migration through the hemolymph included 5 pattern recognition receptors: *9 *antimicrobial effectors, and four prophenoloxidases (Additional file 1). The down-regulation of PPOs associated with sporozoite migration through the hemolymph may represent a host homeostatic mechanism to prevent "toxic shock" following the massive release of these parasite stages into the hemocoel. The remaining immunity-related genes exhibiting significant down-regulation were various and disparate components of the major immunity-related signaling pathways (Additional file 1).

Other notable genes significantly up-regulated during sporozoite passage through the hemolymph included several implicated in phagocytosis (the p41 subunit of the Arp2/3 complex, gelsolin and troponin C) and redox metabolism (a cytochrome P450 and a glutathione S transferase). Additionally, the transcript of an uncharacterized gene (ENSANGT00000032065), encoding a domain with homology to mammalian β-defensin, was significantly up-regulated in hemocytes at day 19 after *P. berghei *infection. The *Drosophila *homolog of this gene has previously been reported to be up-regulated in oncogenic larval hemocytes [80], and it possibly represents a novel antimicrobial peptide induced by, and with activity against, sporozoites.

## Conclusion

We have identified 4047 genes expressed in adult female *An. gambiae *hemocytes, including 959 genes that were differentially expressed following bacterial challenge and/or malaria parasite infection. A dominant proportion of these regulated genes was represented by 105 recognized immunity-related genes, of which many have known or putative roles in defense against *P. berghei *and other species of *Plasmodium*. This pattern is fully consistent with hemocytes having an important role in regulating mosquito innate immune responses. Transcriptomic profiling of *An. gambiae *hemocytes following exposures to various microbes also revealed distinct transcriptomes in response to different species of pathogen and at different stages of infection with the same pathogen. A closer examination of these differential transcriptome signatures provided numerous insights to potentially important functional attributes of hemocyte -mediated defenses. For example, the profound transcriptional response upon challenge with *M. luteus *and the much weaker and mainly down-regulated gene response after *E. coli *challenge, taken together with the reported higher virulence of *E. coli *to *An. gambiae*, suggests that the potency of the immune response activated by these two bacterial species is quite different [27,81].

The distinct transcriptional profiles associated with the two different stages of the malaria parasite infection likely reflects differences in where parasites are located within the mosquito, antigenic differences between ookinetes and sporozoites, and/or temporal differences associated with blood-feeding or age of the mosquito hosts [20,21,60,82]. Of particular interest was the co-regulation in hemocytes of different members of the Imd/REL2 and JNK immune signaling pathways, together with various components of HAT/HDAC multiprotein complexes that regulate immune gene expression through modification of chromatin structure. These gene expression signatures are discussed in Additional file 2, data section S8. The lack of a transcripional phagocytic response to sporozoite infection is in agreement with the recent finding that the rapid disappearance of *P. berghei *sporozoites from the hemolymph of *An. gambiae *apparently results from currently uncharacterized, non-phagocytic and humoral immune mechanisms [20,21,35,61]. Finally, the transcriptomic profiles of hemocytes described in our study revealed that several factors known to influence *Plasmodium *infection are not only induced in the midgut epithelium during ookinete invasion but are also simultaneously up-regulated in hemocytes (e.g. *CLIPB4*, *CLIPB17*, *SRPN6*, and *RFABG*) [29,62,79]. This observation raises questions about the site of action of these immunity-related factors, and whether these molecules have similar or different functions in different tissues. Future challenges, therefore, will be to dissect the contribution of differential gene expression in hemocytes in defense against different species of *Plasmodium*, and to investigate the functional significance of the many novel candidate immunity-related and other genes, identified in this study.

## Methods

### Insects

All experiments were conducted using the G3 strain of *An. gambiae *reared as previously outlined [31].

### Hemocyte counts and phagocytosis/melanization assays

Phagocytosis assays were performed as previously described [31]. Briefly, 2 × 10^3 ^heat-killed. fluoresceing isothiocyanate (FITC)-conugated *E. coli *or *M. luteus *were injected intrathoracically into cold anethetized mosquitoes. After 1 h at room temperature, hemocytes were collected, placed into primary culture, and identified as outlined by Castillo et al (2006) [31]. Briefly, granulocytes were identified by their spread morphology, oenocytoids were identified by morphology, differential labeling with monochlorobimane (MCB) and an anti-phenoloxidase antibody (PP06, generously donated by K. Michel and F. Kafatos), and prohemocytes were identified by morphology and an absence of labeling by MCB and the anti-PO antibody. The proportion of granulocytes that had ingested particles was determined by counting 100 cells in a randomly selected field of view using the fluorescent quenching method [31]. Melanization was quantified by counting hemocyte-internalized bacteria. The proportion of oenocytoids and granulocytes that had melanized without ingesting any bacteria was also determined by visual inspection. We infected mosquitoes with malaria parasites by allowing 4 day-old adult females to feed on a BALB C mouse infected with the PbGFP_CON _strain of *P. berghei*, which constitutively expresses green fluorescent protein (GFP) under control of the *P. berghei *elongation factor 1α promoter [83]. A *Plasmodium berghei *reference line that constitutively expresses GFP at a high level throughout the complete life cycle. Molecular and Biochemical Parasitology [137, [23-33]]. Only mice exhibiting 3–5% parasitemia were used for infection of mosquitoes. Hemocytes were collected either 24 hours or 19 days after blood-feeding. All cohorts of infected mosquitoes were also monitored by dissection to determine levels of midgut infection by oocysts at 24 h and infection of salivary glands by sporozoites on day 19. Only cohorts in which ≥ 80% of individuals were infected were used for analysis. Controls included day 4 mosquitoes with no bleed feeding and mosquitoes blood-fed on uninfected mice. All data were analyzed by ANOVA followed by Dunnetts comparison procedure or t-test using the JMP 7.0 statistical platform (SAS, Gary, NC). Proportional data were arcsin transformed before analysis.

### RNA extraction

Hemocyte samples from bacteria-infected, *P. berghei-*infected and control mosquitoes were prepared and collected as described above. A minimum of 30 individuals were bled and their hemocytes pooled to obtain at least 500 ng of total RNA per replicate. However, for the day 19 post-blood feeding samples, more than 100 mosquitoes were bled per sample due to the reduced number of hemocytes present per individual (see Results). Total RNAs were then isolated from hemocytes using the RNAEasy kit (Qiagen) as outlined by the manufacturer. For each treatment and time point, three independent samples were prepared for use in subsequent microarray and QRT-PCR analyses.

### Microarray probe synthesis, hybridization, analysis and validation

300–400 ng of total RNA was used to synthesize Cy-3 or Cy-5 fluorochrome-labeled cRNA probes for each hemocyte or whole adult female sample using Agilent's Low RNA Input Linear Amplification Kit (Cat. No. 5184-3523; Agilent Technologies, Inc., Wilmington, DE) according to the manufacturer's instructions. Two-color microarray hybridizations were performed using Agilent's In situ Hybridization Kit Plus (Cat. No. 5184-3568) and custom-made 60-mer oligonucleotide microarrays purchased from Agilent as previously reported [64]. Arrays were hybridized for 16 hours, washed, dried with pressurized air and immediately scanned using an Axon 4200AL scanner and GenePix Pro (version 6.0) software (Axon Instruments, Union City, CA). Scanned microarray images were aligned to annotation files and flagged for bad spots in GenePix Pro, using a combination of automatic and manual curation. For our analysis, good spots were defined as expressed if the mean foreground intensity of the spot was at least three standard deviations above the mean local background signal for the same spot. ExpressConverter (version 1.7), MIDAS (version 2.19) and MeV (version 4.0) packages of the TIGR TM4 microarray software suite [84] were used for subsequent downstream analyses of the processed output from GenePix Pro. The array data were normalized with LOWESS and graphically explored using the MIDAS package [85], while significantly differentially expressed transcripts were identified using the significance analysis of microarrays (SAM) [86] feature of the MeV software, with a false discovery rate (FDR) of 5% [87]. Hierarchical clustering was performed with Cluster 3.0 software, using uncentered Pearson correlation distance metric and average linkage clustering method, and the resulting expression clusters visualized using TreeView (version 1.6) software [6,88].

This microarray gene expression platform has been previously validated [64] and we compared real-time quantitative PCR – based expression data to the microarray expression data for four control genes in the 24 hr *P. berghei *challenged and 24 hr non-infected blood fed samples. Total RNA samples were reversed transcribed using dT_20 _primers and Superscript III (Cat. No. 18080-93; Invitrogen, Carlsbad, CA). Real-time quantitative PCR assays were performed using QuantiTect SYBR Green PCR Kit (Cat. No. 204143; Qiagen Inc., Valencia, CA) and ABI Detection System ABI Prism 7000 (Applied Biosystems, Foster City, CA). The ribosomal protein S7 gene was used for normalization of cDNA templates, the specificity of the PCR reactions was confirmed by melting curves analysis. Primer sequences used for microarray validation have been previously published [64]. The expression ratio (infected/non-infected) for the control genes in the microarray (first number) and real-time quantitative PCR (second number) – assays were: DEF1: 0.34, 0.63; CLIPA9: 1.89, 1.71; SRPN9: 3.00, 2.05; AgMDL1: 1.38, 2.18. Minor differences in the magnitude of regulation relate to differences in the sensitivity and dynamic range between the two types of assays, while the direction of regulation was consistent. Microarray data sets have been submitted to GEO: GSM402884/Uninf Bf Hemo vs Pla Hemo 19d C, GSM402883/Uninf Bf Hemo vs Pla Hemo 19d B, GSM402882/Uninf Bf Hemo vs Pla Hemo 19d A, GSM402881/Uninf Bf Hemo vs Pla Hemo 24 hr C, GSM402880/Uninf Bf Hemo vs Pla Hemo 24 hr B, GSM402879/Uninf Bf Hemo vs Pla Hemo 24 hr A, GSM402874/Hemo m luteus vs hemo unchallenge C, GSM402873/Hemo m luteus vs hemo unchallenge B, GSM402872/Hemo m luteus vs hemo unchallenge A, GSM402871/Hemo E coli vs hemo unchallenge C, GSM402870/Hemo E coli vs hemo unchallenge B, GSM402869/Hemo E coli vs hemo unchallenge A, GSM402868/hemo vs whole 2, GSM402867/Hemo vs whole 3, GSM402830/Hemo vs whole.

## List of abbreviations

AMP: antimicrobial peptide; AP-1: adaptor protein complex 1; CEC: cecropin; cfu: colony forming unit; DEF: defensin; FBN: fibrinogen-domain-containing immunolectin; GALE: galectin; GAM: gambicin; GNBP: Gram-negative binding protein; HAT: histone acetyltransferase; HDAC: histone deacetylase; LPS: lipopolysaccharide; LYS: lysozyme; PGRP: peptidoglycan-recognition protein; PO: phenoloxidase; PPO: prophenoloxidase; PPR: pattern recognition receptor; RTQ-PCR: real-time quantitative polymerase chain reaction; TEP: thio-ester containing protein.

## Authors' contributions

LB carried out microarray assays and analyses, and participated in writing the manuscript. AR carried out hemocyte assays. EW carried out microarray assays and analyses. MS conceived of the study, and participated in its design and coordination and participated in writing the manuscript. GD conceived of the study, and participated in its design and coordination, the analysis of data and writing the manuscript. All authors read and approved the final manuscript.

## Supplementary Material

Additional file 1 **Table S1**. Log2 transformed expression ratio microarray data for the various experimental comparisons. Significantly up- and down-regulated genes are indicated with either "Up" or "Down" in separate columns. Click here for file

Additional file 2 **Additional text files S1 – S9**. Additional texts that are complementary to their respective main text sections. Click here for file
