# Supplementary material for: Genome-wide transcriptomic profiling of Anopheles gambiae hemocytes reveals pathogen-specific signatures upon bacterial challenge and Plasmodium berghei infection
Source: BMC Genomics. 2009 Jun 5;10:257. doi: 10.1186/1471-2164-10-257 (PMC2703655; doi:10.1186/1471-2164-10-257)
Supplement: Additional file 2 — Additional text files S1 – S9. Additional texts that are complementary to their respective main text sections. [file 1471-2164-10-257-S2.doc]

**SUPPLEMENTARY MATERIAL**

**[S1]:** Non-immunity -related transcripts significantly enriched in hemocytes were: 13 belonging to the cytoskeletal/structural functional class, including the profilin *chickadee*, which regulates bacterial phagocytosis in *Drosophila* [1, 2]; two uncharacterized serine proteases (ENSANGT00000016466 and ENSANGT00000017208), which were also up-regulated by *M. luteus* challenge and 24 hours after *P. berghei* infection; 4 cytochrome P450s, enzymes involved in detoxification, which have previously been reported to be immune responsive and differentially expressed in mosquito lines with different histories of exposure to malaria parasite infection [3, 4]; 8 enzymes participating in energy metabolism and glycolysis, including hexokinase, 6-phosphofructokinase, 6-phosphofructo-2-kinase, glyceraldehyde 3-phosphate dehydrogenase, phosphoenolpyruvate carboxykinase, and two alcohol dehydrogenases, several of which are induced upon immune challenge in *Drosophila* hemolymph [5]; and 5 putative components of multiprotein complexes known from other systems to regulate immune gene transcription through modification of chromatin structure [6-9]. This latter group included a TIP60-like MYST family histone acetyltransferase (*HAT*), a histone deacetylase (*HDAC*), the actin-like Brahma-associated protein 55kD (*Bap55*), the SWI2/SNF2-like ATPase/helicase domino and enhancer of polycomb (*E(Pc)*). In *Drosophila*, *HAT*, *domino* and *E(Pc)* have previously been shown to influence mycobacterial infection [6]. Phosphatidylethanolamine binding protein (PEBP), augmenter of liver regeneration (ALR) and NADP-dependent leukotriene B4 12-hydroxydehydrogenase (LHDH; EC 1.3.1.74) were also enriched in hemocytes. PEBP is an immune inducible protein in the hemolymph of *Drosophila*, and may regulate NF-κB/MAPK immune signaling pathways through kinase and/or protease inhibition [5, 10]. ALR belongs to a the conserved Erv1/Arl family of growth factors found throughout the eukaryotic kingdom [11]. In mammals, ALR promotes growth of hepatocytes through activation of Kupffer cells, the resident macrophages of the liver [12], and it is possible that ALR derived from hemocytes may have analogous effects on insect fat body cells. ALR was also immune inducible, exhibiting up- and down-regulation, respectively, following challenge with *M. luteus* and 19 days after *P. berghei* infection. LHDH catalyzes the conversion of leukotriene B4, an eicosanoid lipid mediator produced by leukocytes during inflammatory reactions, into its biologically less active metabolite, 12-oxo-leukotriene B4. Eicosanoids have been shown to mediate cellular immune responses to a variety of pathogens in a variety of insects [13].

Some 18 genes predicted to function in chemosensory perception included sensory appendage proteins 1 and 3 (SAP1 and SAP3), which have previously been reported to be expressed in mosquito hemocytes or induced in decapitated adult females following immune stimulation with LPS [14, 15]. The remaining 16 chemosensory-related transcripts detected in hemocytes were annotated as odorant-binding proteins (OBPs) or contained predicted odorant/pheromone-binding domains. Two of these OBP-related transcripts were enriched in hemocytes compared to whole adult females (*OBP2* and *OBP9*), while 6 others were immune-responsive (*OBP5*, *OBP24*, *OBP57*, ENSANGT00000013682, ENSANGT00000013781 and ENSANGT00000025864). The protein products of *OBP9* and the D7-related ENSANT00000013781 have been detected in the hemolymph of adult female *An. gambiae* [16], while genes encoding 4 other OBPs not detected here are differentially transcribed following either bacterial or fungal challenge [3]. In *Drosophila*, 3 different OBPs have also been reported to be present within the hemolymph and induced by bacterial, fungal and/or viral infection [5, 17] consistent with insect OBPs possessing novel immune functions unrelated to their predicted roles in chemosensory perception.

**[S2]:** The lysozyme *LYSC1* [18]; the prophenoloxidases *PPO4* and *PPO6* [19, 20]; the CLIP-domain serine proteases *CLIPB14* and *CLIPB15* [21]; the serine protease inhibitors (serpins) *SRPN6* and *SRPN10* (serpins) [22, 23]; the multidomain-containing scavenger receptor *SCRASP1* (*Sp22D*) [23-25]; the complement-like thio-ester containing protein *TEP1* [26, 27]; and the regulatory subunit of the 26S proteosome *PSMD3* (*DoxA2*) [23]. However, there was only limited expression data for the transcription factor *STAT1* [28], which failed to meet our stringency criteria for inclusion in the final catalog of genes expressed in hemocytes (data not shown). Additionally, we detected transcripts of 22 other genes whose protein products have been detected in the hemolymph of *An. gambiae*, but not previously identified as originating from hemocytes [16, 29-33]. This latter category included the following immunity-related genes: bacteria-responsive protein 1 (*AgBR1*); *PPO2*; *CLIPA6*, *CLIPA8* (inconsistent expression; data not shown), *CLIPB1* (*Sp14D2*), *CLIPB4* (*Sp14D1*), *CLIPB9/10* (*Sp14A*), *CLIPC3* (*Sp18D*); *SRPN1*, *SRPN2* and *SRPN15*; and *TEP15*. A homolog of *CLIPB9/10* is known to be expressed in hemocytes of the mosquito *An. dirus*, a vector of malaria in south-east Asia [34], providing corroboration of the expression of this gene in mosquito hemocytes.

**[S3]:** The immunity-related transcripts enriched in hemocytes included two encoding fibrinogen-domain-containing immunolectins (FBN8 and 9) that have previously been implicated in defense against both bacteria and *Plasmodium* [35], and genes encoding CLIPC7 (ISPR5), inhibitor of apoptosis 2 (IAP2), the homologue of *Drosophila* NimC-1, PPO9, a TOLL interacting protein, and a transferrin were enriched in hemocytes. *PPO9* gave some of the most intense hybridization signals on our microarrays. *PPO9* transcription was also immune responsive, being up- and down-regulated, respectively, by challenge with *M. luteus* and 19 days after *P. berghei* infection. The transferrin represented by ENSANGT00000021949 was enriched in hemocytes, and down-regulated by both bacterial challenges and 24 hours after *P. berghei* infection. This transferrin has previously been reported to be differentially expressed following similar microbial exposures [4, 35], while other transferrins are immune responsive [3, 36] and their protein products present in the hemolymph [5, 36]. The transcript of a novel putative immunity-related gene (ENSANGT00000013469) containing a TNF receptor/CD95 and a protein kinase C/diacylglycerol-binding domain, and predicted to have a role in intracellular signaling, was also enriched in hemocytes. The immunity-related transcripts under-represented in hemocytes included that encoding the c-type lectin CTL4, which protects the ookinetes from being melanizated during their emergence from the midgut epithelium [37, 38]. Transcripts encoding several other pattern recognition receptors (AgMDL2, LRRD8, LRRD20, TEP2 and TEP15) were also under-represented in hemocytes relative to whole adult female mosquitoes.

**[S4]:** Both bacterial species down-regulated *CLIPD1*, *SP CLIP 3*, *FBN8* and the transferrin represented by ENSANGT00000021949. The snake-like serine protease *CLIPC9* (*SP SNAKE-LIKE 1*) was the only transcript up-regulated by infection with *E. coli* and down-regulated by infection with *M. luteus*. CLIPC9 is a paralog of the *Drosophila* extracellular serine protease Persephone, which mediates activation of the Toll signaling pathway in response to fungal infection [39]. *CLIPC9* transcription has previously been reported to be induced in whole mosquitoes following challenge by either *E. coli* or *S. aureus* [35]. The differential pattern of *CLIPC9* expression identified here suggests that this serine protease may be involved in regulating the different transcriptional responses of hemocytes to *E. coli* and *M. luteus*. Immunity-related genes specifically up-regulated transcriptionally following challenge with *M. luteus* included: the AMP *DEF4*; the PRRs *GALE5*, *LRRD20*, *SCRB5*, and *TEP2*; the serine proteases *CLIPB17* and *SP CLIP1*; and *PPO9*. SP CLIP1 is necessary for survival following bacterial challenge [35], but functional studies on the remaining genes have not yet been published.

**[S5]:** RNAi-mediated silencing of *cactus*, a negative regulator of the Toll pathway, which causes constitutive immune activation [6], results in a greater than 2-fold increase in the phagocytosis of *E. coli* [40]. *CED6L*, the *An. gambiae* homologue of an intracellular adaptor protein involved in the removal of apoptotic/necrotic bodies in *Caenorhabditis elegans*, is necessary for efficient phagocytosis of both *E. coli* and the Gram-positive bacterium *Staphylococcus aureus* [40]. In contrast, *PGRPLA*, *PGRPLC* and *TEP3* are required for the phagocytosis of *E. coli* but not *S. aureus* [40] and TEP3 and CED6L are believed to be components of a common pathway mediating phagocytosis of *E. coli* [40]. Co-regulation of both *TEP3* and *CED6L* following *M. luteus* challenge provides further support for this hypothesis but also suggests this pathway is not specific for Gram-negative bacteria.

**[S6]:** Tyrosine hydroxylase (TH; also known as tyrosine 3-monooxygenase; EC 1.14.16.2; ENSANGT00000012551); a dopachrome conversion enzyme (DCE; EC 4.1.1.28; ENSANGT00000013424); an uncharacterized hemocyanin (ENSANGT00000016795); and LYSC2. The transcript encoding TH was significantly induced by *M. luteus*, while transcripts of DCE, hemocyanin and LYSC2 were specifically up-regulated by *E. coli*. In the mosquito *Armigeres subalbatus* DCE is expressed in hemocytes, up-regulated upon filarial infection, and facilitates melanization of filarial worms [41]. Hemocyanins are primarily known for their function in oxygen transport through the hemolymph [42]. Increasing evidence implicates hemocyanins, which are structurally and functionally related to POs, in immune defense and melanization [43, 44]. In *An. gambiae*, the hemocyanin (HECY1) is necessary for protection against infection with *S. aureus* [35]. The function of lysozymes in mosquito innate immune responses is currently not well-understood, although another *LYSC1* regulates melanization of foreign bodies [32]. *LYSC2* has previously been reported to be induced in the thorax of adult *An. gambiae* following bacterial challenge [45].

**[S7]:** *E. coli* either did not induce or down-regulated genes with putative roles in phagocytosis, while *M. luteus* up-regulated several phagocytosis-associated genes. For example, 11 of the 12 differentially expressed genes belonging to the cytoskeletal/structural functional class were down-regulated by challenge with *E. coli*, while 15 of the 18 genes differentially expressed following challenge with *M. luteus* were up-regulated. Among the transcripts up-regulated by *M. luteus* were: adhesion regulating molecule 1 (ARP1); afadin, an actin filament-binding protein; two ARFs; the p30 subunit of the Arp2/3 complex; α- and β-integrins; moesin; several myosins; a tropomyosin and a troponin; a shroom homologue; and rho1 and rhoL small GTPases as well as various other rho-related factors. The *An. gambiae* genome encodes two β-integrins (BINT1 and BINT2), of which only *BINT2* has previously been reported to influence phagocytosis of *E. coli* [40, 46]. Although both β-integrins were expressed in hemocytes, only *BINT1* was differentially expressed, following challenge with *M. luteus*, as were two putative autophagy pathway proteins (ENSANT00000002536 and ENSANT00000016064). The role of autophagy in phagocytosis of pathogens is increasingly being recognized [47, 48]. The enzyme *β*-hexosaminidase was also transcriptionally down-regulated by challenge with *M. luteus*. In *Drosophila* and mammalian systems, *β*-hexosaminidase is released from lyzosomes during phagocytic internalization of bacteria and possesses a peptidoglycan hydrolase activity controlling mycobacterial infection [49]. Although no functional data are currently available for *An. gambiae*, *β*-hexosaminidase transcription has previously been reported to be induced in adult females by inoculation of LPS [14].

**[S8]:** Functional studies in *Drosophila* indicate that IAP2, TAK1 and IKK2 mediate Imd/Relish-dependent immune responses [50-56], although the role of TAK1 is currently controversial [7, 57]. Regardless, in *Drosophila*, cross-regulation between the Imd/Relish and JNK signaling pathways is well-established [50-52, 57-59], with JNK-mediated signaling transcriptionally regulating Imd/Relish-dependent immune responses through binding of the transcription factor AP-1 to the promoters of NF-κB target genes and subsequent recruitment of chromatin-modifying HAT/HDAC complexes [7, 60]. The AP-1 transcription factor is a heterodimer comprised of two interacting partners: c-Jun and Fos (in *Drosophila* also known, respectively, as Jun-related antigen and kayak [61]). Both AP-1 partners were expressed in hemocytes from *An. gambiae*, and c-Jun was specifically up-regulated at 24 hours after *P. berghei* infection. Additionally, two other transcripts encoding the HAT and the HDAC enriched in hemocytes were differentially regulated during the period of ookinete invasion of the midgut epithelium (Figure S8). Interestingly, some of the Imd/REL2 and/or HAT/HDAC components described above were also differentially regulated by bacterial challenge: *E. coli* regulated *IMD* and *IKK2* in the opposite, and *M. luteus* regulated *IAP2*, *HAT* and *HDAC* in the same, direction to 24 hours after *P. berghei* infection (Figure S8). The latter observation may explain the partially overlapping transcriptomic signatures of hemocytes following *M. luteus* challenge and 24 hours after *P. berghei* infection (Figure S8).

**Figure S8.** Coordinated transcriptional regulation in *An. gambiae* hemocytes of the Imd/REL2 and JNK immune signaling pathways, and HAT/HDAC complex members, during the period of *P. berghei* ookinete invasion of the midgut epithelium. Red and green color indicate up- and down-regulation, respectively, and black indicate no regulation between the compared samples: Hemocyte versus whole, *E. coli* challenged versus non-challenges, *M. luteus* challenged versus non-challenged, *P. berghei* ookinete infected at 24 hours after infected blood meal versus non-infected blood meal fed, *P. berghei* sporozoite infected at 19 days after infected blood meal versus non-infected blood meal fed.


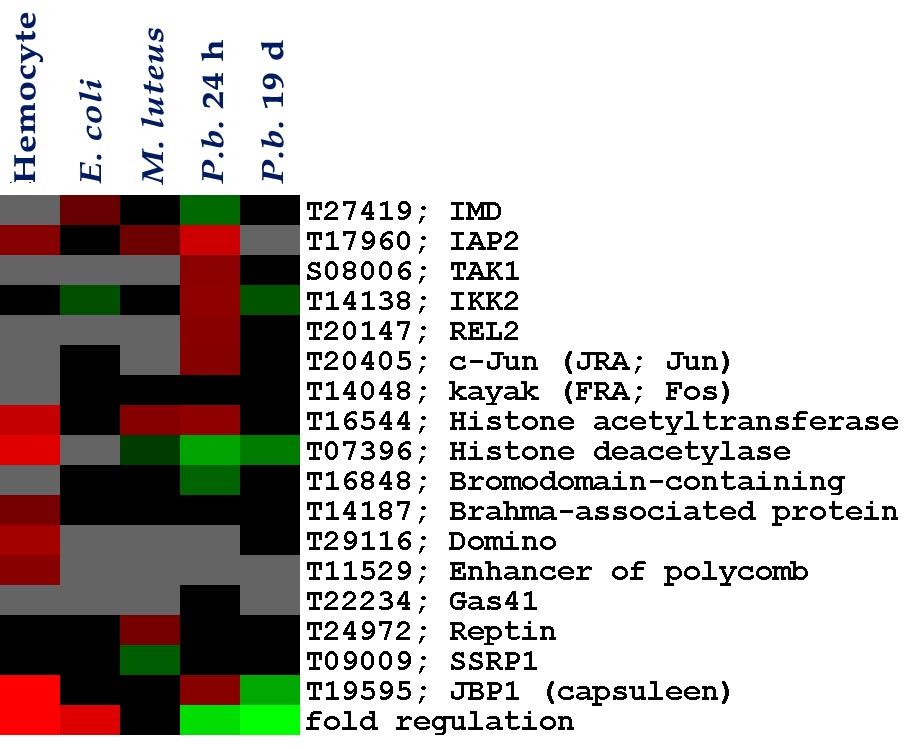


**[S9]:** The co-regulation of this subset of CLIP-domain serine proteases and their serpin inhibitors suggests that they comprise a module of interacting partners operating during ookinete invasion of the midgut epithelium. CLIPA4, CLIPA6 and CLIPA9 have no role in melanization of *P. berghei* ookinetes in the susceptible G3 strain of *An. gambiae* used in our studies. In contrast, CLIPA7 has previously been reported to inhibit melanization of *P. berghei* ookinetes [38]. CLIPB4 promotes the melanization of foreign bodies like Sephadex beads [62], and both CLIPB4 and CLIPB17 promote melanization of *P. berghei* ookinetes in the refractory L3-5 strain of *An. gambiae* [38]. However, neither CLIPB4 nor CLIPB17 affect the melanization of *P. berghei* ookinetes in the G3 strain of *An. gambiae*, unless expression of the protective antagonist of melanization CTL4 is abolished [38]. In addition, an uncharacterized CLIPA-like serine protease encoded by transcript ENSANGT00000020158 was down-regulated in hemocytes 24 hours after *P. berghei* infection, while three uncharacterized serine proteases (ENSANGT00000014761, ENSANGT00000021238 and ENSANGT00000031509), exhibiting bloodmeal-specific expression, were up-regulated. SRPN6 and SRPN10 have previously been reported to be specifically up-regulated in ookinete-invaded midgut epithelial cells, but are also known to be expressed in hemocytes [22, 23, 63]. Functional studies implicate SRPN6 in anti-*Plasmodium* defense during ookinete invasion of the midgut epithelium [22], while SRPN10 is believed to mediate apoptosis of ookinete-invaded midgut epithelial cells [64, 65]. SRPN6 has been proposed to inhibit melanization of *P. berghei* ookinetes in *An. gambiae* [22], and elevated levels of this serpin may partly explain the absence of ookinete melanization in the G3 strain despite the presumably melanizing-promoting transcript levels of *CLIPA7*, *CLIPB4* and *CLIPB17*. The function of SRPN10 in hemocytes is unknown: hemocyte numbers did not vary during *P. berghei* infection, and our gene expression data revealed no signature of apoptosis, implying that hemocytes do not undergo apoptosis during malaria parasite infection. SRPN10 might have a different function in hemocytes, or its function in midgut epithelial cells may not be related to apoptosis. *SRPN10* has been reported to influence phagocytosis of bacteria (Supplementary Material in [40]). The role of the third up-regulated serpin (*SRPN17*) during *Plasmodium* infection has not been investigated.

**References**

1. Pearson AM, Baksa K, Ramet M, Protas M, McKee M, Brown D, Ezekowitz RA: **Identification of cytoskeletal regulatory proteins required for efficient phagocytosis in *Drosophila***. *Microbes Infect* 2003, **5**(10):815-824.

2. Philips JA, Rubin EJ, Perrimon N: ***Drosophila* RNAi screen reveals CD36 family member required for mycobacterial infection**. *Science* 2005, **309**(5738):1251-1253.

3. Aguilar R, Jedlicka AE, Mintz M, Mahairaki V, Scott AL, Dimopoulos G: **Global gene expression analysis of *Anopheles gambiae* responses to microbial challenge**. *Insect Biochem Mol Biol* 2005, **35**(7):709-719.

4. Aguilar R, Das S, Dong Y, Dimopoulos G: **Continuous exposure to *Plasmodium* results in decreased susceptibility and transcriptomic divergence of the *Anopheles gambiae* immune system**. *BMC Genomics* 2007, **8**:451.

5. Levy F, Bulet P, Ehret-Sabatier L: **Proteomic analysis of the systemic immune response of *Drosophila***. *Mol Cell Proteomics* 2004, **3**(2):156-166.

6. Agaisse H, Burrack LS, Philips JA, Rubin EJ, Perrimon N, Higgins DE: **Genome-wide RNAi screen for host factors required for intracellular bacterial infection**. *Science* 2005, **309**(5738):1248-1251.

7. Kim LK, Choi UY, Cho HS, Lee JS, Lee WB, Kim J, Jeong K, Shim J, Kim-Ha J, Kim YJ: **Down-regulation of NF-kappaB target genes by the AP-1 and STAT complex during the innate immune response in *Drosophila***. *PLoS Biol* 2007, **5**(9):e238.

8. Li B, Samanta A, Song X, Iacono KT, Bembas K, Tao R, Basu S, Riley JL, Hancock WW, Shen Y *et al*: **FOXP3 interactions with histone acetyltransferase and class II histone deacetylases are required for repression**. *Proc Natl Acad Sci U S A* 2007, **104**(11):4571-4576.

9. Sapountzi V, Logan IR, Robson CN: **Cellular functions of TIP60**. *Int J Biochem Cell Biol* 2006, **38**(9):1496-1509.

10. Vierstraete E, Verleyen P, Baggerman G, D'Hertog W, Van den Bergh G, Arckens L, De Loof A, Schoofs L: **A proteomic approach for the analysis of instantly released wound and immune proteins in *Drosophila* *melanogaster* hemolymph**. *Proc Natl Acad Sci U S A* 2004, **101**(2):470-475.

11. Gatzidou E, Kouraklis G, Theocharis S: **Insights on augmenter of liver regeneration cloning and function**. *World J Gastroenterol* 2006, **12**(31):4951-4958.

12. Wang CP, Zhou L, Su SH, Chen Y, Lu YY, Wang F, Jia HJ, Feng YY, Yang YP: **Augmenter of liver regeneration promotes hepatocyte proliferation induced by Kupffer cells**. *World J Gastroenterol* 2006, **12**(30):4859-4865.

13. Stanley D: **Prostaglandins and other eicosanoids in insects: biological significance**. *Annu Rev Entomol* 2006, **51**:25-44.

14. Oduol F, Xu J, Niare O, Natarajan R, Vernick KD: **Genes identified by an expression screen of the vector mosquito *Anopheles gambiae* display differential molecular immune response to malaria parasites and bacteria**. *Proc Natl Acad Sci U S A* 2000, **97**(21):11397-11402.

15. Bartholomay LC, Cho WL, Rocheleau TA, Boyle JP, Beck ET, Fuchs JF, Liss P, Rusch M, Butler KM, Wu RC *et al*: **Description of the transcriptomes of immune response-activated hemocytes from the mosquito vectors *Aedes* *aegypti* and *Armigeres subalbatus***. *Infect Immun* 2004, **72**(7):4114-4126.

16. Paskewitz SM, Shi L: **The hemolymph proteome of *Anopheles gambiae***. *Insect Biochem Mol Biol* 2005, **35**(8):815-824.

17. Sabatier L, Jouanguy E, Dostert C, Zachary D, Dimarcq JL, Bulet P, Imler JL: **Pherokine-2 and -3**. *Eur J Biochem* 2003, **270**(16):3398-3407.

18. Baton LA, Ranford-Cartwright LC: **Spreading the seeds of million-murdering death: metamorphoses of malaria in the mosquito**. *Trends Parasitol* 2005, **21**(12):573-580.

19. Hillyer JF, Barreau C, Vernick KD: **Efficiency of salivary gland invasion by malaria sporozoites is controlled by rapid sporozoite destruction in the mosquito haemocoel**. *Int J Parasitol* 2007, **37**(6):673-681.

20. Blandin S, Shiao SH, Moita LF, Janse CJ, Waters AP, Kafatos FC, Levashina EA: **Complement-like protein TEP1 is a determinant of vectorial capacity in the malaria vector *Anopheles gambiae***. *Cell* 2004, **116**(5):661-670.

21. Volz J, Osta MA, Kafatos FC, Muller HM: **The roles of two clip domain serine proteases in innate immune responses of the malaria vector *Anopheles gambiae***. *J Biol Chem* 2005, **280**(48):40161-40168.

22. Abraham EG, Pinto SB, Ghosh A, Vanlandingham DL, Budd A, Higgs S, Kafatos FC, Jacobs-Lorena M, Michel K: **An immune-responsive serpin, SRPN6, mediates mosquito defense against malaria parasites**. *Proc Natl Acad Sci U S A* 2005, **102**(45):16327-16332.

23. Castillo JC, Robertson AE, Strand MR: **Characterization of hemocytes from the mosquitoes *Anopheles gambiae* and *Aedes* *aegypti***. *Insect Biochem Mol Biol* 2006, **36**(12):891-903.

24. Danielli A, Loukeris TG, Lagueux M, Muller HM, Richman A, Kafatos FC: **A modular chitin-binding protease associated with hemocytes and hemolymph in the mosquito *Anopheles gambiae***. *Proc Natl Acad Sci U S A* 2000, **97**(13):7136-7141.

25. Gorman MJ, Andreeva OV, Paskewitz SM: **Sp22D: a multidomain serine protease with a putative role in insect immunity**. *Gene* 2000, **251**(1):9-17.

26. Blandin SA, Levashina EA: **Phagocytosis in mosquito immune responses**. *Immunol Rev* 2007, **219**:8-16.

27. Frolet C, Thoma M, Blandin S, Hoffmann JA, Levashina EA: **Boosting NF-kappaB-dependent basal immunity of *Anopheles gambiae* aborts development of *Plasmodium* *berghei***. *Immunity* 2006, **25**(4):677-685.

28. Barillas-Mury C, Han YS, Seeley D, Kafatos FC: ***Anopheles gambiae* Ag-STAT, a new insect member of the STAT family, is activated in response to bacterial infection**. *Embo J* 1999, **18**(4):959-967.

29. Gorman MJ, Paskewitz SM: **Persistence of infection in mosquitoes injected with bacteria**. *J Invertebr Pathol* 2000, **75**(4):296-297.

30. Shi L, Paskewitz SM: **Identification and molecular characterization of two immune-responsive chitinase-like proteins from *Anopheles gambiae***. *Insect Mol Biol* 2004, **13**(4):387-398.

31. Michel K, Budd A, Pinto S, Gibson TJ, Kafatos FC: ***Anopheles gambiae* SRPN2 facilitates midgut invasion by the malaria parasite *Plasmodium* *berghei***. *EMBO Rep* 2005, **6**(9):891-897.

32. Li B, Paskewitz SM: **A role for lysozyme in melanization of Sephadex beads in *Anopheles gambiae***. *J Insect Physiol* 2006, **52**(9):936-942.

33. Schnitger AK, Kafatos FC, Osta MA: **The melanization reaction is not required for survival of *Anopheles gambiae* mosquitoes after bacterial infections**. *J Biol Chem* 2007, **282**(30):21884-21888.

34. Xu W, Huang FS, Hao HX, Duan JH, Qiu ZW: **Two serine proteases from *Anopheles dirus* haemocytes exhibit changes in transcript abundance after infection of an incompatible rodent malaria parasite, *Plasmodium* yoelii**. *Vet Parasitol* 2006, **139**(1-3):93-101.

35. Dong Y, Aguilar R, Xi Z, Warr E, Mongin E, Dimopoulos G: ***Anopheles gambiae* immune responses to human and rodent *Plasmodium* parasite species**. *PLoS Pathog* 2006, **2**(6):e52.

36. Yoshiga T, Hernandez VP, Fallon AM, Law JH: **Mosquito transferrin, an acute-phase protein that is up-regulated upon infection**. *Proc Natl Acad Sci U S A* 1997, **94**(23):12337-12342.

37. Osta MA, Christophides GK, Kafatos FC: **Effects of mosquito genes on *Plasmodium* development**. *Science* 2004, **303**(5666):2030-2032.

38. Volz J, Muller HM, Zdanowicz A, Kafatos FC, Osta MA: **A genetic module regulates the melanization response of Anopheles to *Plasmodium***. *Cell Microbiol* 2006, **8**(9):1392-1405.

39. Ligoxygakis P, Pelte N, Hoffmann JA, Reichhart JM: **Activation of *Drosophila* Toll during fungal infection by a blood serine protease**. *Science* 2002, **297**(5578):114-116.

40. Moita LF, Wang-Sattler R, Michel K, Zimmermann T, Blandin S, Levashina EA, Kafatos FC: **In vivo identification of novel regulators and conserved pathways of phagocytosis in *A. gambiae***. *Immunity* 2005, **23**(1):65-73.

41. Huang CY, Christensen BM, Chen CC: **Role of dopachrome conversion enzyme in the melanization of filarial worms in mosquitoes**. *Insect Mol Biol* 2005, **14**(6):675-682.

42. Burmester T, Hankeln T: **The respiratory proteins of insects**. *J Insect Physiol* 2007, **53**(4):285-294.

43. Cerenius L, Soderhall K: **The prophenoloxidase-activating system in invertebrates**. *Immunol Rev* 2004, **198**:116-126.

44. Jiang N, Tan NS, Ho B, Ding JL: **Respiratory protein-generated reactive oxygen species as an antimicrobial strategy**. *Nat Immunol* 2007, **8**(10):1114-1122.

45. Li B, Calvo E, Marinotti O, James AA, Paskewitz SM: **Characterization of the c-type lysozyme gene family in *Anopheles gambiae***. *Gene* 2005, **360**(2):131-139.

46. Moita LF, Vriend G, Mahairaki V, Louis C, Kafatos FC: **Integrins of *Anopheles gambiae* and a putative role of a new beta integrin, BINT2, in phagocytosis of *E. coli***. *Insect Biochem Mol Biol* 2006, **36**(4):282-290.

47. Levine B: **Unraveling the role of autophagy in cancer**. *Autophagy* 2006, **2**(2):65-66.

48. Sanjuan MA, Dillon CP, Tait SW, Moshiach S, Dorsey F, Connell S, Komatsu M, Tanaka K, Cleveland JL, Withoff S *et al*: **Toll-like receptor signalling in macrophages links the autophagy pathway to phagocytosis**. *Nature* 2007, **450**(7173):1253-1257.

49. Koo IC, Ohol YM, Wu P, Morisaki JH, Cox JS, Brown EJ: **Role for lysosomal enzyme beta-hexosaminidase in the control of mycobacteria infection**. *Proc Natl Acad Sci U S A* 2008, **105**(2):710-715.

50. Silverman N, Zhou R, Erlich RL, Hunter M, Bernstein E, Schneider D, Maniatis T: **Immune activation of NF-kappaB and JNK requires *Drosophila* TAK1**. *J Biol Chem* 2003, **278**(49):48928-48934.

51. Park JM, Brady H, Ruocco MG, Sun H, Williams D, Lee SJ, Kato T, Jr., Richards N, Chan K, Mercurio F *et al*: **Targeting of TAK1 by the NF-kappa B protein Relish regulates the JNK-mediated immune response in *Drosophila***. *Genes Dev* 2004, **18**(5):584-594.

52. Valanne S, Kleino A, Myllymaki H, Vuoristo J, Ramet M: **Iap2 is required for a sustained response in the *Drosophila* Imd pathway**. *Dev Comp Immunol* 2007, **31**(10):991-1001.

53. Gesellchen V, Kuttenkeuler D, Steckel M, Pelte N, Boutros M: **An RNA interference screen identifies Inhibitor of Apoptosis Protein 2 as a regulator of innate immune signalling in *Drosophila***. *EMBO Rep* 2005, **6**(10):979-984.

54. Kleino A, Valanne S, Ulvila J, Kallio J, Myllymaki H, Enwald H, Stoven S, Poidevin M, Ueda R, Hultmark D *et al*: **Inhibitor of apoptosis 2 and TAK1-binding protein are components of the *Drosophila* Imd pathway**. *Embo J* 2005, **24**(19):3423-3434.

55. Leulier F, Lhocine N, Lemaitre B, Meier P: **The *Drosophila* inhibitor of apoptosis protein DIAP2 functions in innate immunity and is essential to resist gram-negative bacterial infection**. *Mol Cell Biol* 2006, **26**(21):7821-7831.

56. Huh JR, Foe I, Muro I, Chen CH, Seol JH, Yoo SJ, Guo M, Park JM, Hay BA: **The *Drosophila* inhibitor of apoptosis (IAP) DIAP2 is dispensable for cell survival, required for the innate immune response to gram-negative bacterial infection, and can be negatively regulated by the reaper/hid/grim family of IAP-binding apoptosis inducers**. *J Biol Chem* 2007, **282**(3):2056-2068.

57. Delaney JR, Stoven S, Uvell H, Anderson KV, Engstrom Y, Mlodzik M: **Cooperative control of *Drosophila* immune responses by the JNK and NF-kappaB signaling pathways**. *Embo J* 2006, **25**(13):3068-3077.

58. Boutros M, Agaisse H, Perrimon N: **Sequential activation of signaling pathways during innate immune responses in *Drosophila***. *Dev Cell* 2002, **3**(5):711-722.

59. Zhuang ZH, Sun L, Kong L, Hu JH, Yu MC, Reinach P, Zang JW, Ge BX: ***Drosophila* TAB2 is required for the immune activation of JNK and NF-kappaB**. *Cell Signal* 2006, **18**(7):964-970.

60. Kim T, Yoon J, Cho H, Lee WB, Kim J, Song YH, Kim SN, Yoon JH, Kim-Ha J, Kim YJ: **Downregulation of lipopolysaccharide response in *Drosophila* by negative crosstalk between the AP1 and NF-kappaB signaling modules**. *Nat Immunol* 2005, **6**(2):211-218.

61. Ciapponi L, Bohmann D: **An essential function of AP-1 heterodimers in *Drosophila* development**. *Mech Dev* 2002, **115**(1-2):35-40.

62. Paskewitz SM, Andreev O, Shi L: **Gene silencing of serine proteases affects melanization of Sephadex beads in *Anopheles gambiae***. *Insect Biochem Mol Biol* 2006, **36**(9):701-711.

63. Danielli A, Kafatos FC, Loukeris TG: **Cloning and characterization of four *Anopheles gambiae* serpin isoforms, differentially induced in the midgut by *Plasmodium* *berghei* invasion**. *J Biol Chem* 2003, **278**(6):4184-4193.

64. Danielli A, Barillas-Mury C, Kumar S, Kafatos FC, Loukeris TG: **Overexpression and altered nucleocytoplasmic distribution of Anopheles ovalbumin-like SRPN10 serpins in *Plasmodium*-infected midgut cells**. *Cell Microbiol* 2005, **7**(2):181-190.

65. Kumar S, Gupta L, Han YS, Barillas-Mury C: **Inducible peroxidases mediate nitration of anopheles midgut cells undergoing apoptosis in response to *Plasmodium* invasion**. *J Biol Chem* 2004, **279**(51):53475-53482.
